# The burden of illness in initiating intermittent catheterization: an analysis of German health care claims data

**DOI:** 10.1186/s12894-021-00814-7

**Published:** 2021-04-08

**Authors:** Almuth Angermund, Gary Inglese, Jimena Goldstine, Laura Iserloh, Berit Libutzki

**Affiliations:** 1 Department of Neuro-Urology, Schön Clinic, Vogtareuth, Germany; 2 Hollister Incorporated, 2000 Hollister Drive, Libertyville, IL 60048-3781 USA; 3 HGC Healthcare Consultants GmbH, Düsseldorf, Germany; 4 Department of Psychiatry, Interdisciplinary Center Psychopathology and Emotion Regulation (ICPE), University of Groningen, University Medical Center Groningen, Groningen, The Netherlands

**Keywords:** Urinary incontinence, Infections, urinary tract, Retrospective study, Continence care products

## Abstract

**Background:**

Intermittent catheterization (IC) is a common medical technique to drain urine from the bladder when this is no longer possible by natural means. The objective of this study was to evaluate the standard of care and the burden of illness in German individuals who perform intermittent catheterization and obtain recommendations for improvement of care.

**Methods:**

A descriptive study with a retrospective, longitudinal cohort design was conducted using the InGef research database from the German statutory health insurance claims data system. The study consisted of individuals with initial IC use in 2013–2015.

**Results:**

Within 3 years 1100 individuals with initial IC were identified in the database (~ 19,000 in the German population). The most common IC indications were urologic diseases, spinal cord injury, Multiple Sclerosis and Spina Bifida. Urinary tract infections (UTI) were the most frequent complication occurring 1 year before index (61%) and in follow-up (year 1 60%; year 2 50%). Resource use in pre-index including hospitalizations (65%), length of stay (12.8 ± 20.0 days), physician visits (general practitioner: 15.2 ± 29.1), prescriptions of antibiotics (71%) and healthcare costs (€17,950) were high. Comorbidities, complications, and healthcare resource use were highest 1 year before index, decreasing from first to second year after index.

**Conclusions:**

The data demonstrated that prior to initial catheterization, IC users experienced UTIs and high healthcare utilization. While this demonstrates a potential high burden of illness prior to initial IC, UTIs also decreased over time, suggesting that IC use may have a positive influence. The findings also showed that after the first year of initial catheterization the cost decreased. Further studies are needed to better understand the extent of the burden for IC users compared to non-IC users.

**Supplementary Information:**

The online version contains supplementary material available at 10.1186/s12894-021-00814-7.

## Background

In the early 1970’s Jack Lapides published on the use of clean intermittent catheterization (IC) and frequent voiding patterns to achieve bladder health [1]. Today, IC is a common medical technique to drain urine from the bladder. The catheterization can be performed by the individuals themselves, referred to as intermittent self-catheterization (ISC), or alternatively by caregivers. IC can be applied either for short term bladder-management or as a long-term solution. If the bladder is not emptied regularly, permanent damage to the bladder and kidneys and infections may be caused [2]. Therefore, IC is generally performed multiple times daily. IC is considered the “gold standard” for medical bladder emptying for individuals with bladder retention and is recommended for individuals with lower urinary tract dysfunction or neurological conditions leading to urological conditions [3].

Multiple Sclerosis (MS), spinal cord injury (SCI) and Spina Bifida (SB) are the more common neurological conditions, and the underactive bladder is the predominant urological indication for the implication of IC. IC may improve the incontinence, but it is not a treatment for this [4]. The correct use of intermittent catheterization and strict compliance with hygiene instructions should avoid negative effects of continuous long-term catheterization, however, still a major complication of catheterization is the increased risk of developing a urinary tract infection (UTI). Other common complications can be urethral strictures, bladder stones or other infections [5–7]. To counteract and/or prevent UTIs, a common therapy is antibiotics, which are prescribed for acute and prophylactic use [8].

The objective of this study was to evaluate the standard of care and the burden of illness in German individuals who perform IC. We are among the first who investigate the comprehensive patient pathway of patients who perform IC. In order to evaluate the state of the current care situation, demographic data, indications, comorbidities, complications and critical events, therapeutic measures and cost dynamics were mapped for a period of 1 year before and 2 years after initial IC. This study provides real-world evidence on IC use, which may be used to derive recommendations for improvement of care in this cohort.

## Construction and content

## Study design and participants

A descriptive study with a retrospective, longitudinal cohort design was conducted obtaining claims data from the InGef research database containing approx. 5 million member-records from over 60 (from a total of 118) nationwide statutory health insurances (SHIs). This equals a 5%-sample of the German population with a projection factor of 16.86 (2012–2017: 81,654,166 total German population/ 4,844,101 patients in database). The analysis was performed at the InGef—Institute for Applied Health Research Berlin GmbH.

Approximately 90% of the German population is insured in SHIs, hence these sources of data are highly representative of the care reality in Germany. All data are anonymized before entering the database. The sample is representative of the German population in terms of age and sex and is widely used for real-world evaluation [9]. The study followed the guidelines of “Good Practice Secondary Data” [10].

**Fig. 1:**
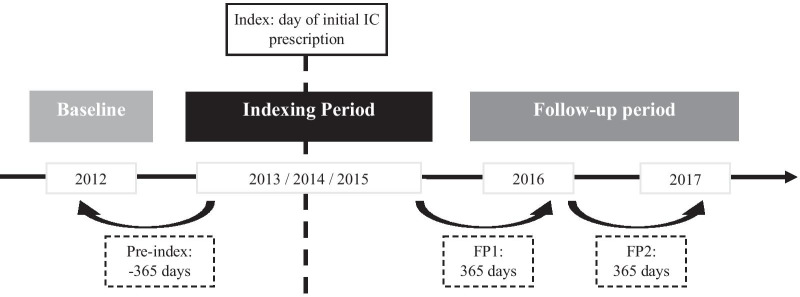
Study design

Data was available from 2012 to 2017. Individuals with initial IC use were identified between January 2013 and December 2015, with the date of IC prescription (German medical aid list 15.25.14*) referred to as the index day. Baseline was 12 months (365 days) before index. Total follow-up period was 24 months (divided in 2 years of follow-up (FP): FP1 and FP2). To ensure initial IC use, individuals with IC prescriptions prior to index (minimum 365 days) were excluded from the analysis. Individuals not continuously insured were excluded from the analysis to avoid missing data and loss to follow-up. Also excluded were individuals with unspecific coding and individuals with more than one IC prescription at index. Individuals who died during the follow-up period were included in the analysis and observed until day of death (Fig. 1).

### Outcomes

To describe the SHI claims data study population basic demographic information (age, sex, mortality, indications for IC use) was extracted for all identified individuals. Indications for IC (based on an ICD-10 GM diagnosis) were: Parkinson, MS, stroke, SB, SCI, other injuries affecting the spinal cord, other causes of paralysis and urologic diseases (various incontinences: stress, reflex, overflow, urge, extraurethrale, recurrence, unspecified; urinary retention, anuria and oligory, polyuria). Various ICD-10-GM codes were summarized to build the specific indication groups—see Additional file 1: Appendix. Outcomes in baseline and follow-up period measured were: comorbidities and complications, pre-defined critical events, therapy modalities including prescription of pre-defined medication and catheters, physician visits, hospitalizations and readmissions. Specific groups per outcome were also build here based on different code summaries and/or combinations—see Additional file 1: Appendix. In addition, direct healthcare costs, sickness benefits and sick leave days were observed.

Office-based physicians were classified according to their medical specialty using the “Arztgruppenschlüssel (AGS)”. “GP” was used for physicians practicing as general practitioners based on AGS 1, 2, 3, 34. “Psychotherapy” was used for physicians practicing as psychiatrists and medical psychotherapists based on AGS 51, 53, 58, 61 and 68. Comorbidities and complications, indications and critical events were identified based on the International Statistical Classification of Diseases and Related Health Problems, 10^th^ Revision, German Modification (ICD-10-GM) corresponding to the specific inpatient primary or secondary or outpatient secured diagnosis in the quarter of index.

Medical aids including specific catheters were identified using chapter 15.25* of the German medical aid list. Remedies, such as physiotherapy are listed within the ‘Heilmittelkatalog’. Outpatient medication were identified based on prescriptions, which are documented at the day the prescription is handed in at the pharmacy. Medication is documented based on the anatomical-therapeutic-chemical classification system (ATC). Procedures according to IC were identified via the catalogue for outpatient services, the “Einheitlicher Bewertungsmaßstab” (EBM) (see Additional file 1).

Healthcare costs were reported for the following categories: total healthcare costs, inpatient, outpatient, medical aids and remedies, medication, sickness benefit and sick days. In Germany, sickness benefits funded by the SHI are available after more than 6 weeks of inability to work. The amount of sickness benefits is calculated based on the regular income. The analysis was descriptive for all outcomes and reported using frequencies and percentages for categorical variables, counts, means, medians, 1st quartile and 3rd quartile and standard deviations (SD) for continuous variable. Data protection requirements established by the board of SHIs prevented the reporting of data from a sample size < 5 (other than 0) and were marked as such. For data storage and processing, Microsoft Office Excel® 2010 (Microsoft Corporation, WA, USA) and SAS® (Version 9.2; SAS Institute Inc., NC, USA) were used.

## Results

### Study population, demography, comorbidities, complications and critical events

**Fig. 2:**
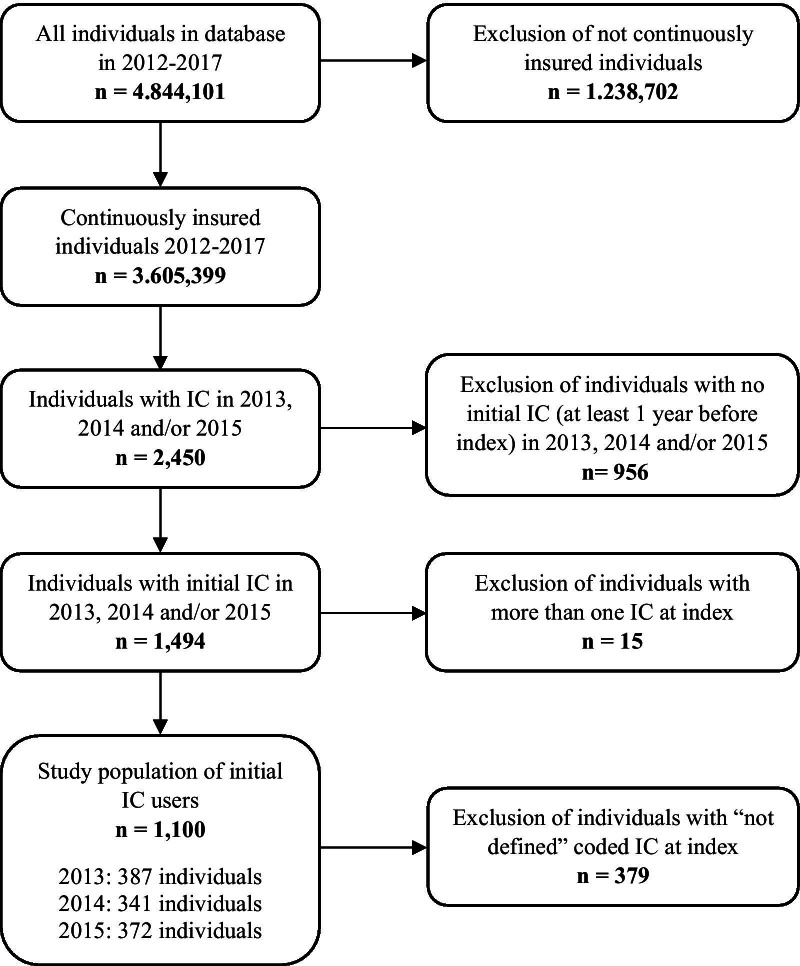
Patient flow

Within the analyses 2450 individuals with initial IC use were found in the indexing period 2013 to 2015 (Fig. 2). After excluding IC use before index (n = 956), 15 individuals with multiple IC prescriptions at index and 379 individuals with unspecific coding, 1100 individuals with initial IC use remained. Projected to the German population this means there were 18,846 individuals initially using IC in Germany within 3 years. The number of initial IC users was evenly distributed over the years at approx. 370 individuals each year, which is about 6238 projected to German population.

Males made up 46% of the study population. On average IC users were 57 years old, the oldest IC user was 98 years old, the youngest not yet 1 year. During the 2-year observation period 12% of the study population died (130 out of 1100 all-cause mortality), mostly within FP1. The most common IC indications were urologic diseases at 47%, which included prostate/bladder/kidney diseases, followed by SCI at 16% and other injuries affecting the spinal cord, like para-/tetraparesis, hemiparesis/-plegia and myelopathy at 12%. Further indications were MS (10%), other causes of paralysis (6%) including cerebral palsy and similar, spina bifida (4%), stroke (4%) and Parkinson’s Disease (3%) (Table 1).

**Table 1:** Demographics/characteristics of IC patients at index

	IC total
Total, n (%)	1100
Male, n (%)	511 (46)
Age in years, mean ± standard derivation	57.3 ± 20.9
Mortality, n (%)*	130 (12)
Indications, n (%)**	
Urologic diseases	516 (47)
Spinal Cord Injury	180 (16)
Other injuries affecting the spinal cord	134 (12)
Multiple Sclerosis	107 (10)
Other causes of paralysis	63 (6)
Stroke	40 (4)
Spina Bifida	45 (4)
Parkinson	30 (3)

Indications for intermittent catheterization (IC) based on diagnosis in follow-up year 1 (FP1) (inpatient primary/secondary or outpatient secured diagnosis). (double count possible—15 patients have more than one diagnosis)

*All-cause mortality

**Indication groups based on ICD-10 codes—see Additional file 1: Appendix

Common comorbidities and complications in pre-index were urologic diseases (87%), UTI (61%), other infections that are not related with the urinary tract (34%), catheter related complications (30%) and other urinary infections (14%) (see Additional file 1). The prevalence of comorbidities and complications was highest in pre-index; comparing pre- and post-index the occurrence of comorbidities and complications decreased by around 10% each. Similarly, critical events were highly prevalent prior to index (58%) and decreased post-index (47%). Before initial IC use, half of the individuals had an UTI diagnosis in combination with at minimum one outpatient or inpatient urologic procedure, including urethroscopy, urine examination and other diagnostic measures. Half of the cohort had a UTI diagnosis in combination with at minimum one prescription of antibiotics; 40% received antibiotics prophylactically. Before initial IC use, 22% of the individuals experiencing non-urinary tract infections received antibiotics and 10% prophylactic antibiotics; the prevalence decreased by 5% during follow-up. Approximately every tenth individual had documentation of the ICD-10 diagnosis code; antibiotic resistance (Table 2).

**Table 2:** Number of patients with specific comorbidities, complications and critical events per year

	PRE-INDEX	POST-INDEX	
	Pre-Index	FP 1	FP 2
IC Total, n	1100	1100	1025
Comorbidities and complications, n (%)*			
Urologic diseases	962 (87)	935 (85)	840 (82)
UTI	669 (61)	662 (60)	515 (50)
Other infections	375 (34)	365 (33)	253 (25)
Catheter related complications*	325 (30)	303 (28)	232 (23)
Other urinary infections	153 (14)	130 (12)	101 (10)
Urethral bleeding	119 (11)	82 (7)	77 (8)
Urinary stricture	18 (2)	21 (2)	8 (1)
Critical events, n (%)**			
UTI & antibiotics	548 (50)	544 (49)	398 (39)
UTI & prophylactic antibiotics	440 (40)	428 (39)	290 (28)
UTI & antibiotic resistance	82 (7)	67 (6)	39 (4)
UTI & fever	32 (3)	39 (4)	20 (2)
UTI & urologic procedure	580 (53)	560 (51)	431 (42)
UTI & any of the above	633 (58)	619 (56)	481 (47)
Other infection & antibiotics	245 (22)	251 (23)	180 (18)
Other infection & prophylactic antibiotics	166 (15)	172 (16)	112 (11)

Comorbidities, complications and critical events based on specific inpatient primary/secondary or outpatient secured diagnosis. Patients are initiating IC at index, however it is possible they have had indwelling catheters or other therapies in the pre-index period. Critical events: diagnosis of UTI/ other infection and in the same quarter one of the combinations. Prophylactic antibiotics: prescriptions in at least two quarters of the same year

*IC* intermittent catheterization, *FP* follow-up, *UTI* urinary tract infection

*Comorbidities/complication groups based on ICD-10 codes—see additional file 1: Appendix

**Critical events based on combinations of ICD-10 codes and/or ATC codes and/or EBM codes—see Additional file 1: Appendix

### Therapies

In pre-index and FP1 92% of the IC users received at least one prescription of medication (Table 3). In FP2, prescription rates decreased marginally. In pre-index the majority (71%) received at least one prescription of antibiotics; prophylactic antibiotics were given to 48% of the IC users. Around two thirds received medication to treat functional disorders of the bladder including anticholinergics, phosphodiesterase inhibitors and similar, 41% pain medication and 24% antidepressants. In FP2 the prescription rate decreased marginally or remained stable. About every fifth IC user continuously obtained antibiotics and/or pain medication in each quarter of the same year. Two years after initial IC use, 50% of the individuals still had IC prescriptions (average usage time 334 days). IC users received around seven IC prescription during the follow-up period (approx. one prescription per quarter).

**Table 3:** Number of patients with specific therapies per year

	PRE-INDEX	POST-INDEX	
	Pre-index	FP 1	FP 2
Total, n	1100	1100	1025
Medication: at least one prescription of specific medication, n (%)*			
Total	1010 (92)	1010 (92)	916 (89)
Antibiotics	783 (71)	809 (74)	670 (65)
Medication for functional disorder of the bladder	686 (62)	674 (61)	564 (55)
Prophylactic antibiotics	533 (48)	575 (52)	424 (41)
Pain medication	452 (41)	474 (43)	424 (41)
Antidepressants	266 (24)	288 (26)	255 (25)
Muscle relaxants	135 (12)	102 (9)	113 (11)
Supplements & herbal anti-infectives	105 (10)	120 (11)	92 (9)
Sleep aids	72 (7)	87 (8)	69 (7)
Sterile rinsing of the bladder	35 (3)	43 (4)	28 (3)
Continuous medication: at least one prescription of specific medication in each quarter of the same year, n (%)			
Antibiotics	122 (11)	168 (15)	118 (12)
Pain medication	112 (10)	131 (12)	112 (11)
Total disjunct	239 (22)	297 (27)	228 (22)
IC catheter prescription, n (%)**			
IC catheter	–	1100 (100)	525 (48)
Mean number of IC prescription/ IC user	–	4.22	5.81

Prophylactic antibiotics: prescriptions in at least two quarters of the same year. Supplements & herbal anti-infectives are OTCs

*IC* intermittent catheterization, *FP* follow-up period, *OTC* over-the-counter-drug

*Medication groups based on ATC codes—see Additional file 1: Appendix

**Based on medical aid number 15.25.14—see Additional file 1: Appendix

Hospitalization rates were highest in pre-index at 65%; 18% were hospitalized due to a urologic disease, 5% due to UTI and 3% because of another infection (see Additional file 1). 41% were re-hospitalized for a second stay within the same year. The average length of stay was 13 days regarding all stays and eight days regarding UTI. Comparing pre- and post-index, hospitalizations and re-admissions decreased by around 20%, the average length of stay decreased by 4.4 days regarding all stays and by 1.1 days regarding UTI. The GP was most frequently contacted healthcare professional, followed by the urologist. A GP was visited on average 15.7 times per year, a urologist 5.2 and a psychotherapist 2.5 times (Table 4).

**Table 4:** Number of patients with hospitalizations and readmissions per year//physicians

	PRE-INDEX	POST-INDEX	
	Pre-Index	FP 1	FP 2
Total, n	1100	1100	1025
Hospitalizations, n (%)			
Total	711 (65)	558 (51)	445 (43)
Due to catheter related complications (inpatient primary diagnosis)*			
Urologic diseases	203 (18)	121 (11)	76 (7)
UTI	56 (5)	56 (5)	29 (2)
Other infections	36 (3)	45 (4)	24 (2)
Readmission, n (%)			
Total	454 (41)	295 (27)	224 (22)
Due to a specific reason*			
Urologic diseases	163 (15)	89 (8)	55 (5)
UTI	48 (4)	41 (4)	24 (2)
Other infections	31 (3)	34 (3)	17 (2)
Length of stay in days, mean ± standard derivation (SD)/median (med)			
Mean ± SD/med	12.8 ± 20.0/7	8.0 ± 8.0/6	8.4 ± 11.7 /5.3
Length of stay due to UTI, ± standard derivation			
Mean ± SD/med	7.8 ± 6.5/6	5.4 ± 2.8/5	6.7 ± 5.5/5
Physician visits, n (%)**			
General practitioner	1077 (98)	1077 (98)	996 (97)
Urologist	742 (67)	780 (71)	653 (64)
Psychotherapist	365 (33)	392 (36)	343 (33)
Specific outpatient physician contacts per individual, mean ± standard derivation (SD)/median (med)			
General practitioner	15.2 ± 29.1/6	16.4 ± 31.1/7	15.6 ± 29.5/7
Urologist	5.3 ± 7.0/3	5.9 ± 7.0/4	4.5 ± 5.6/3
Psychotherapist	2.4 ± 10.5/0	2.5 ± 11.3/0	2.4 ± 10.1/0

Patients are initiating intermittent catheterization (IC) at index, however it is possible they have had indwelling catheters or other therapies in the pre-index period

*FP* follow-up, *UTI* urinary tract infection

*Groups based on ICD-10 codes—see Additional file 1: Appendix

**Physicians based on AGS codes—see Additional file 1: Appendix

### Costs

Total healthcare costs per individual and year ranged between ~ €18,000 and €22,000 with a peak visible in the year of initial IC use. Cost drivers were inpatient costs ~ €6000 to €11,000, aids and remedies ~ €2000 to €11,000 and medication at ~ €3500. Inpatient costs and sick pay decreased by more than half comparing pre- and post-index; sick days decreased by 7.5 days. Costs for aids and remedies more than quintupled comparing pre- and post-index. Medication and outpatient costs including antibiotics and pain medication remained consistent over time (Table 5).

**Table 5:** Average costs per IC user per year

	PRE-INDEX	POST-INDEX	
	Pre-Index	FP 1	FP 2
Total, n	1100	1100	1025
Overall healthcare costs in € per individual			
Outpatient sector	1355	1390	1351
Inpatient sector	10,738	6943	4370
Due to emergency stays	3646	2529	1623
Due to UTI*	225	121	111
ER stay due to UTI* (primary diagnosis)	146	75	49
Medication**	3298	3509	3165
Pain medication	141	171	174
Antibiotics	57	60	47
Medical aids and remedies	2063	9949	11,036
Sick pay (sick days)	496 (17.3)	627 (17.6)	247 (9.8)
Total healthcare costs***	17,950	22,418	20,168

*FP* follow-up, *UTI* urinary tract infection, *ER* emergency room

*based on ICD-10 codes—see Additional file 1: Appendix

**Based on ATC codes—see Additional file 1: Appendix

***Average amount (€) of health care costs per IC user

## Discussion

### Discussion of findings

We are among the first to study a 3-year observation period including time before and after initial IC. Moreover, as studies on IC with larger samples sizes are rare, we present highly relevant findings to depict the reality of care in IC users. Our study is consistent with other published literature describing the profile of IC users [11, 12]. The relative prevalence of the urologic diseases represented in our dataset are similar to that reported in the guideline for management and implementation of IC [26] and other published studies [13, 27]. Notably, several diagnoses in the group of urologic diseases stem from conditions such as SCI, MS and/or spina bifida. Despite the prevalence of these chronic conditions, our data also found that every second user stopped IC after 1 year. This is consistent with other published studies where the recovery of the bladder function was a common reason for stopping IC [11].

The most common complication evident in IC users was recurrent UTIs, which is considered a severe complication [2, 13]. The highest prevalence of comorbidities, complications, and critical events, including UTI, was recorded before initial IC use, which may suggest that patients are experiencing inadequate bladder management prior to initiating IC. UTI rates decreased when comparing pre- and post-index, which is in line with previously published hypotheses that IC does not necessarily lead to UTI and may have a positive impact on UTIs overtime [2, 6, 14]. This positive impact is further emphasized by the decrease of complications during FP1 and FP2. IC is also recommended by the National Institute of Health and Care Excellence (NICE) which claims IC reduces the risk of UTIs and maintains bladder health [15].

The high illness burden was also visible in elevated hospitalization rates, length of stay and readmission rates. The main reasons for hospitalization were urologic diseases and UTI. UTIs have been shown to increase the number of hospital admissions and length of stay [7]. 13% of the German population (compared to 50% of IC users in our study) have at least one hospital stay per year [16] and stay for on average of 7.3 days (compared to 10 days of IC users in our study) [17].

The main contact physician was the GP, followed by the urologist. Individuals who perform IC were associated with a mean of 16 GP visits per year. Approximately one third visited a psychologist per year (before and after initial IC use). While the data does not describe the reasons for those visits, this underscores additional cost burden to the healthcare system.

The daily life of IC users was influenced by the prescription of many drugs. Medication for functional disorder of the bladder includes anticholinergics, which are usually given to paraplegic patients, leading to a reduction of the contractility of the detrusor [18]. Comparing our data to the overall German population, prescription rates of antibiotics are particularly high (30% German population vs. on average 70% IC users in our database) [19]. While prophylactic antibiotics are associated with a reduction of the frequency of UTI, they are also associated with increasing antimicrobial resistance [8]. Bacteria in urine are often immediately misidentified as UTI and treated with antibiotics, regardless of suitability and possible resistances [20]. Thus, antibiotic therapy should only be used in case of symptomatic or clinically relevant UTI [26]. Further education regarding the benefits and risks associated with prescribing antibiotics is important given emergence of resistant urinary pathogens as a public health concern [21].

The high illness burden is also reflected in that IC users are incurring on average approximately €20,000 per year in healthcare spend. This is compared to the 2018 average health costs per SHI member in Germany were at €4200 [22]. Overall, we found that healthcare costs remained relatively stable with an increase at the year of initial IC use but a decrease in FP2. While costs of aids and remedies increase, inpatient costs, sick pay and sick days decreased during follow-up, suggesting that individuals using IC found ways to successfully manage and address their clinical complications. Medication costs including costs for pain medication and antibiotics mostly levelled off overtime and continue for the disease management. These findings are supported by studies that demonstrate that using IC led to cost reductions due to lower complication-rates and use of healthcare resources [2]. Several studies have further demonstrated how IC is associated with positive health and quality of life outcomes. IC has been shown to promote an individual’s independence, preserve his/her dignity and reduces embarrassment. IC allows people to partake in leisure activities, and gives freedom from obstructive devices, and helps improve/maintain sexual/intimate relationships [14, 23].

The data suggests that IC users start with a high burden of illness. Overtime, while they can successfully manage their condition, it comes at a cost to the healthcare system. Thus, to mitigate those costs and further support better outcomes, IC users should have continued access to products and therapies that best meet their unique bladder management needs [2]. Böthig et al. state, it is not medically justified to limit the frequency of catheterization, product type or product access due to the economic burden [26]. Thus, IC users should have access to physician specialists, like neuro-urologists, and specialized care centers who will tailor their care to each individual. Further research is needed to better understand this patient population and explore more deeply into the causes that underlie their high burden of illness prior to initial intermittent catheterization.

### Strength and limitations

This study’s major strength is the availability of data before and after initial IC and a large sample size. The multitude of available endpoints within this representative sample of the German population provides valuable insights into the reality of care and costs as experienced by individuals with IC. Approximately 90% of the German population is insured in SHIs, hence these sources of data are highly representative of the care reality in Germany. The sample is representative of the German population in terms of age and sex and is widely used for real-world evaluation [9]. SHI claims data analysis is an established procedure in health care research and internationally recognized [9].

This retrospective study is based on SHI claims data, which is recorded for the primary purpose of billing. Hence, this source of data is limited in terms of primary information by physicians and individuals themselves and does not depict costs that are paid out-of-pocket or not SHI-born [24]. Furthermore, the database does not differentiate between individuals performing of self IC vs. those who need assistance. Research focusing on the implications between self and assisted IC is necessary. Moreover, the database does not specify the quantity of catheters that were prescribed per patient. As such, prescriptions are representative only of patient access to product. The data collected represents results for Germany and treatment patterns and costs may differ in other countries, therefore the generalizability of the data is unknown. Moreover, this is a database of healthcare claims, actual costs to the healthcare system may be lower if there are contractual payment agreements. However, this study provides directional insights to the economic burden within the healthcare system. As with all real-world registries, the data presented is dependent on the quality and completeness of the data available [25]. Finally, due to the retrospective nature of the study, we cannot make any correlations of causation, only associations.

### Conclusion

The data demonstrated that prior to initial catheterization, IC users experienced high healthcare utilization. Moreover, IC users showed a high burden of illness even before initial catheterization as indicated by comorbidities and complications such as UTIs. However, UTIs also decreased over time which suggests that IC technique may have a positive influence. The findings also showed that after a peak in the initial year of catheterization, healthcare costs decreased again in the second year of follow-up. Further studies are needed to further understand the extend of the burden for IC users compared to non-IC users.

## Supplementary Information

**Additional file 1: Appendix**. The appendix clusters our subgroups and shown with codes are included in the respective group.

## Data Availability

The datasets analysed during the current study are available from the Institute for Applied Health Research Berlin (InGef) (ed.fegni@ofni), on reasonable request.

## Abbreviations

AGS: Arztgruppenschlüssel
ATC: Anatomical-therapeutic-chemical-classification system
EBM: Einheitlicher Bewertungsmaßstab
ER: Emergency room
FP: Follow-up
GP: General practitioner
IC: Intermittent catheterization
ICD-10(-GM): International Statistical Classification of Diseases and Related health Problems, 10th Revision, (German Modification)
ICPE: Interdisciplinary Center Psychopathology and Emotion regulation
InGef: Institute for Applied Health Research Berlin GmbH
ISC: Intermittent self-catheterization
OTC: Over-the-counter-drug
MS: Multiple Sclerosis
NICE: National Institute of Health and Care Excellence
Psych: Psychotherapy
SB: Spina Bifida
SCI: Spinal cord injury
SD: Standard derivation
SHI: Statutory health insurance
