# Supplementary material for: The burden of illness in initiating intermittent catheterization: an analysis of German health care claims data
Source: BMC Urol. 2021 Apr 8;21:57. doi: 10.1186/s12894-021-00814-7 (PMC8028779; doi:10.1186/s12894-021-00814-7)
Supplement: Supplementary file 1 — Additional file 1: Appendix. The appendix clusters our subgroups and shown with codes are included in the respective group. [file 12894_2021_814_MOESM1_ESM.docx]

**Appendix**

| **Inclusion/Exclusion: Identification of individuals with IC based on at least one prescription of IC medical aids during index** | | |
| --- | --- | --- |
| **Medical aid number.** | | **Description** |
| 15.25.14* | | Single-use catheter for ISC |
| **Codes used in table 1** | | |
| **Indications for IC based on diagnosis in FP1 (inpatient primary/secondary or outpatient secured diagnosis)** | | |
| **ICD-10 GM** | **Description** | |
| Parkinson | | |
| G20* | Primary Parkinson Syndrome | |
| G21* | Secondary Parkinson Syndrome | |
| MS | | |
| G35* | Multiple Sclerosis | |
| Stroke | | |
| G45* | Cerebral transitory ischemia and related syndromes | |
| I63* | Cerebral infarction | |
| I64 | Stroke, not referred to as bleeding or infarction | |
| Spina Bifida | | |
| Q05* | Spina Bifida | |
| Spinal Cord Injury | | |
| G82* | Paraparesis and paraplegia, tetraparesis and tetraplegia | |
| G82.0* | Flaccid paraparesis and paraplegia | |
| G82.00 | Flaccid paraparesis and paraplegia: Acute complete paraplegia of nontraumatic origin | |
| G82.01 | Flaccid paraparesis and paraplegia: Acute incomplete paraplegia of nontraumatic origin | |
| G82.02 | Flaccid paraparesis and paraplegia: Chronic complete paraplegia | |
| G82.03 | Flaccid paraparesis and paraplegia: Chronic incomplete paraplegia | |
| G82.09 | Flaccid paraparesis and paraplegia: unspecified | |
| G82.1* | Spastic paraparesis and paraplegia | |
| G82.10 | Spastic paraparesis and paraplegia: Acute complete paraplegia of nontraumatic origin | |
| G82.11 | Spastic paraparesis and paraplegia: Acute incomplete paraplegia of nontraumatic origin | |
| G82.12 | Spastic paraparesis and paraplegia: Chronic complete paraplegia | |
| G82.13 | Spastic paraparesis and paraplegia: Chronic incomplete paraplegia | |
| G82.19 | Spastic paraparesis and paraplegia: unspecified | |
| G82.2* | Paraparesis and paraplegia, unspecified | |
| G82.20 | Paraparesis and paraplegia, unspecified: Acute complete paraplegia of nontraumatic origin | |
| G82.21 | Paraparesis and paraplegia, unspecified: Acute incomplete paraplegia of nontraumatic origin | |
| G82.22 | Paraparesis and paraplegia, unspecified: Chronic complete paraplegia | |
| G82.23 | Paraparesis and paraplegia, unspecified: Chronic incomplete paraplegia | |
| G82.29 | Paraparesis and paraplegia, unspecified: unspecified | |
| G82.3* | Flaccid tetraparesis and tetraplegia | |
| G82.30 | Flaccid tetraparesis and tetraplegia: Acute complete paraplegia of nontraumatic origin | |
| G82.31 | Flaccid tetraparesis and tetraplegia: Acute incomplete paraplegia of nontraumatic origin | |
| G82.32 | Flaccid tetraparesis and tetraplegia: Chronic complete paraplegia | |
| G82.33 | Flaccid tetraparesis and tetraplegia: Chronic incomplete paraplegia | |
| G82.39 | Flaccid tetraparesis and tetraplegia: unspecified | |
| G82.4* | Spastic tetraparesis and tetraplegia | |
| G82.40 | Spastic tetraparesis and tetraplegia: Acute complete paraplegia of nontraumatic origin | |
| G82.41 | Flaccid tetraparesis and tetraplegia: Acute incomplete paraplegia of nontraumatic origin | |
| G82.42 | Flaccid tetraparesis and tetraplegia: Chronic complete paraplegia | |
| G82.43 | Flaccid tetraparesis and tetraplegia: Chronic incomplete paraplegia | |
| G82.49 | Spastic tetraparesis and tetraplegia: unspecified | |
| G82.5* | Tetraparesis and tetraplegia, unspecified | |
| G82.50 | Tetraparesis and tetraplegia, unspecified: Acute complete paraplegia of nontraumatic origin | |
| G82.51 | Tetraparesis and tetraplegia, unspecified: Acute incomplete paraplegia of nontraumatic origin | |
| G82.52 | Tetraparesis and tetraplegia, unspecified: Chronic complete paraplegia | |
| G82.53 | Tetraparesis and tetraplegia, unspecified: Chronic incomplete paraplegia | |
| G82.59 | Tetraparesis and tetraplegia, unspecified: unspecified | |
| G82.6*! | Functional level of spinal cord damage | |
| G82.60! | C1-C3 | |
| G82.61! | C4-C5 | |
| G82.62! | C6-C8 | |
| G82.63! | T1-T6 | |
| G82.64! | T7-T10 | |
| G82.65! | T11-L1 | |
| G82.66! | L2-S1 | |
| G82.67! | S2-S5 | |
| G82.69! | Unspecified | |
| P11* | Other birth injuries of the central nervous system | |
| P11.0 | Brain edema due to childbirth injury | |
| P11.1 | Other specified brain damage due to childbirth injury | |
| P11.2 | Unspecified brain damage due to childbirth injury | |
| P11.3 | Birth injury of the facial nerve [VII. cranial nerve] | |
| P11.4 | Birth injury to other cranial nerves | |
| P11.5* | Birth injury of the spine and spinal cord | |
| P11.50 | With acute paraplegia | |
| P11.51 | With chronic paraplegia | |
| P11.59 | Unspecified | |
| P11.9 | Birth injury of the central nervous system, unspecified | |
| S14* | Injury of the nerves and the spinal cord at neck height | |
| S14.0 | Contusion and oedema of the cervical spinal cord | |
| S14.1* | Other and unspecified cervical spinal cord injuries | |
| S14.10 | Injuries of the cervical spinal cord, unspecified | |
| S14.11 | Complete cross-sectional injury of the cervical spinal cord | |
| S14.12 | Central neck mark injury (incomplete cross section injury) | |
| S14.13 | Other incomplete cross-sectional injuries of the cervical spinal cord | |
| S14.2 | Injury of nerve roots of the cervical spine | |
| S14.3 | Injury of the brachial plexus | |
| S14.4 | Injury of peripheral nerves of the neck | |
| S14.5 | Injury of cervical sympathetic nerves | |
| S14.6 | Injury of other and unspecified nerves of the neck | |
| S14.7*! | Functional height of a cervical spinal cord injury | |
| S14.70! | Size not specified | |
| S14.71! | C1 | |
| S14.72! | C2 | |
| S14.73! | C3 | |
| S14.74! | C4 | |
| S14.75! | C5 | |
| S14.76! | C6 | |
| S14.77! | C7 | |
| S14.78! | C8 | |
| S24* | Injury of the nerves and spinal cord at thorax level | |
| S24.0 | Contusion and oedema of the thoracic spinal cord | |
| S24.1* | Other and unspecified injuries of the thoracic spinal cord | |
| S24.10 | Injuries of the thoracic spinal cord, unspecified | |
| S24.11 | Complete cross-sectional injury of the thoracic spinal cord | |
| S24.12 | Incomplete cross-sectional injury of the thoracic spinal cord | |
| S24.2 | Injury of nerve roots of the thoracic spine | |
| S24.3 | Injury of peripheral nerves of the thorax | |
| S24.4 | Injury of thoracic sympathetic nerves | |
| S24.5 | Injury of other nerves of the thorax | |
| S24.6 | Injury to an unspecified nerve of the thorax | |
| S24.7*! | Functional height of a thoracic spinal cord injury | |
| S24.70! | Size not specified | |
| S24.71! | T1 | |
| S24.72! | T2/T3 | |
| S24.73! | T4/T5 | |
| S24.74! | T6/T7 | |
| S24.75! | T8/T9 | |
| S24.76! | T10/T11 | |
| S24.77! | T12 | |
| S34* | Injury of the nerves and the lumbar spinal cord at the level of the abdomen, the lumbosacral region and the pelvis | |
| S34.0 | Contusion and oedema of the lumbar spinal cord [Conus medullaris] | |
| S34.1* | Other injuries of the lumbar spinal cord | |
| S34.10 | Complete cross-sectional injury of the lumbar spinal cord | |
| S34.11 | Incomplete cross-sectional injury of the lumbar spinal cord | |
| S34.18 | Other injuries of the lumbar spinal cord | |
| S34.2 | Injury of nerve roots of the lumbar spine and sacrum | |
| S34.3* | Injury of the Cauda equina | |
| S34.30 | Complete traumatic cauda (equina) syndrome | |
| S34.31 | Incomplete traumatic cauda (equina) syndrome | |
| S34.38 | Other and unspecified injuries to Cauda equina | |
| S34.4 | Injury of the plexus lumbosacralis | |
| S34.5 | Injury of sympathetic nerves of the lumbar vertebrae, sacrum and pelvic region | |
| S34.6 | Injury to one or more peripheral nerves of the abdomen, lumbosacral region and pelvis. | |
| S34.7*! | Functional height of a lumbosacral spinal cord injury | |
| S34.70! | Size not specified | |
| S34.71! | L1 | |
| S34.72! | L2 | |
| S34.73! | L3 | |
| S34.74! | L4 | |
| S34.75! | L5 | |
| S34.76! | S1 | |
| S34.77! | S2-S5 | |
| S34.8 | Injury of other and unspecified nerves at the level of the abdomen, the lumbosacral region and the pelvis | |
| Other injuries affecting the spinal cord | | |
| G83* | Other paralysis syndromes | |
| G83.0 | Diparesis and diplegia of the upper extremities | |
| G83.1 | Monoparesis and monoplegia of a lower extremity | |
| G83.2 | Monoparesis and monoplegia of an upper extremity | |
| G83.3 | Monoparesis and monoplegia, unspecified | |
| G83.4* | Cauda- (equina-) syndrome | |
| G83.40 | Complete cauda- (equina-) syndrome | |
| G83.41 | Incomplete cauda- (equina-) syndrome | |
| G83.49 | Cauda- (equina-) syndrome, unspecified | |
| G83.5 | Locked-in-syndrome | |
| G83.8 | Other specified paralysis syndromes | |
| G83.9 | Paralysis syndrome, unspecified | |
| G95* | Other diseases of the spinal cord | |
| G95.0 | Syringomyellia and Syringobulbia | |
| G95.1* | Vascular myelopathies | |
| G95.10 | Non-traumatic spinal hemorrhage | |
| G95.18 | Other vascular myelopathies | |
| G95.2 | Spinal cord compression, unspecified | |
| G95.8* | Other specified diseases of the spinal cord | |
| G95.80 | Urinary bladder paralysis with damage to the upper motor neuron | |
| G95.81 | Urinary bladder paralysis with damage to the lower motor neuron | |
| G95.82 | Urinary bladder dysfunction due to spinal shock | |
| G95.83 | Spinal spasticity of the striated musculature | |
| G95.84 | Detrusor-Sphinkter dyssynergy in spinal cord damage | |
| G95.85 | Deafferentiation pain in spinal cord damage | |
| G95.88 | Other specified diseases of the spinal cord | |
| G95.9 | Diseases of the spinal cord, unspecified | |
| Other causes of paralysis | | |
| G80* | Infantile cerebral palsy | |
| G80.0 | Spastic tetraplegic cerebral palsy | |
| G80.1 | Spastic diplegic cerebral palsy | |
| G80.2 | Infantile hemiplegic cerebral palsy | |
| G80.3 | Dyskinetic cerebral palsy | |
| G80.4 | Atactic cerebral palsy | |
| G80.8 | Other infantile cerebral palsy | |
| G80.9 | Infantile cerebral palsy, unspecified | |
| G81* | Hemiparesis and hemiplegia | |
| G81.0 | Flaccid hemiparesis and hemiplegia | |
| G81.1 | Spastic hemiparesis and hemiplegia | |
| G81.9 | Hemiparesis and hemiplegia, unspecified | |
| Urologic diseases | | |
| N39.3 | Stress incontinence | |
| N39.4* | Other specified urinary incontinence | |
| N39.40 | Reflex incontinence | |
| N39.41 | Overflow incontinence | |
| N39.42 | Urge incontinence | |
| N39.43 | Extraurethral urinary incontinence | |
| N39.47! | Recurrence incontinence | |
| N39.48 | Other Harninkontinenz | |
| R32 | Unspecified urinary incontinece | |
| R33 | urinary retention | |
| R34 | Anuria and oligory | |
| R35 | Polyuria | |
| **Codes used in table 2** | | |
| **Comorbidites, complications and critical events (specific inpatient primary/secondary or outpatient secured diagnosis) + combinations (ICD-10 and ICD-10; ICD-10 and ATC; ICD-10 and EBM)** | | |
| **ICD-10 GM** | **Description** | |
| UTI (Urinary tract infections) | | |
| N30.0 | Acute cystitis | |
| N30.1 | Interstitial cystitis (chronic) | |
| N30.2 | Other chronic cystitis | |
| N30.9 | Cystitis unspecified | |
| N34.1 | Unspecific urethritis | |
| N34.2 | Other urethritis | |
| N37.0 | Urethritis in diseases classified elsewhere | |
| N39.0 | Urinary tract infection, localization not further described | |
| Other urinary infections | | |
| N00* | Acute nephritic syndrome | |
| N01* | Rapid-progressive nephritic syndrome | |
| N03* | Chronic nephritic syndrome | |
| N05* | Unspecified nephritic syndrome | |
| N10 | Acute tubulointerstitial nephritis | |
| N11* | Chronic tubulointerstitielle Nephritis | |
| N12 | Tubulointerstitielle Nephritis, not described as acute or chronic | |
| N30.3 | Trigonum cystitis | |
| N30.4 | Radiation cystitis | |
| N30.8 | Other cystitis | |
| N34.0 | Urethral abscess | |
| N34.3 | Urethral syndrome, unspecified | |
| N39.8* | Other specified urinary incontinence | |
| N39.81 | Flank pain hematuria syndrome | |
| N39.88 | Other specified diseases of the urinary system | |
| N39.9 | Diseases of the urinary system, unspecified | |
| N41* | Inflammatory diseases of the prostate gland | |
| O23* | Infections of the urogenital tract during pregnancy | |
| R82.7 | Abnormal findings during microbiological urinalysis | |
| Catheter related complications | | |
| A40* | Streptococcal sepsis | |
| A41* | Other Sepsis | |
| D41* | Reproduction of insecure or unknown behaviour of the urinary organs | |
| N02* | Recurrent and persistent hematuria | |
| N10* | Acute tubulointerstitial nephritis | |
| N11* | Chronic tubulointerstitielle Nephritis | |
| N21* | Stone in the lower urinary tract | |
| N34.0 | Urethral abscess | |
| N35* | Urethral stricture | |
| N36.0 | Urethral fistula | |
| N36.1 | Diverticulum of the urethra | |
| N39.3 | Stress incontinence | |
| N39.4* | Other specified urinary incontinence | |
| N39.40 | Reflex incontinence | |
| N39.41 | Overflow incontinence | |
| N39.42 | Urge incontinence | |
| N39.43 | Extraurethral urinary incontinence | |
| N39.47! | Recurrence incontinence | |
| N39.48 | Other incontinence | |
| N39.81 | Flank pain hematuria syndrome | |
| N99.1 | Urethral stricture after medical measures | |
| R31 | Unspecified hematuria | |
| R32 | Unspecified urinary incontinece | |
| S37* | Injury of the urinary and pelvic organs | |
| Other infections | | |
| A39.2 | Acute meningococcal sepsis | |
| A39.3 | Chronic meningococcal sepsis | |
| A39.4 | Meningococcal sepsis, unspecified | |
| A40* | Streptococcal sepsis | |
| A41* | Other Sepsis | |
| A48.8 | Other specified bacterial diseases | |
| A49* | Bacterial infection of unspecified localization | |
| B37.4 | Candidosis at other sites of the urogenital system | |
| B37.7 | Candida sepsis | |
| B95-*! | Streptococci and staphylococci as causes of diseases classified in other chapters | |
| B96-*! | Other specified bacteria as the cause of diseases classified in other chapters | |
| B96.2! | Escherichia coli [E. coli] and other enterobacteriaceae as cause of diseases classified in other chapters | |
| J12* | Virus pneumonia, not elsewhere classified | |
| J13* | Pneumonia caused by Streptococcus pneumoniae | |
| J14* | Pneumonia caused by Haemophilus influenzae | |
| J15* | Pneumonia caused by bacteria, not classified elsewhere | |
| J16* | Pneumonia caused by other infectious agents, not elsewhere classified | |
| J17* | Pneumonia in diseases classified elsewhere | |
| J18* | Pneumonia, pathogen not described in more detail | |
| I33* | Acute and subacute endocarditis | |
| N45* | Orchitis and epidimytitis | |
| R50* | Fever of other and unknown causes | |
| R65.-* | Systemic inflammatory response syndrome [SIRS] | |
| U80!* | Pathogens with certain antimicrobial resistance requiring special therapeutic or hygienic measures | |
| U80!* | Gram-positive pathogens with certain antimicrobial resistance requiring special therapeutic or hygienic measures | |
| U81! | Bacteria with multiresistance to antibiotics | |
| U81!* | Gram-negative pathogens with certain antimicrobial resistance requiring special therapeutic or hygienic measures | |
| Urologic diseases | | |
| C61 | Malignant neoplasm of the prostate gland | |
| C66 | Malignant neoplasm of the ureter | |
| C67* | Malignant neoplasm of the urinary bladder | |
| C68* | Malignant neoplasm of other and unspecified urinary organs | |
| D29.1 | Benign prostatic neoplasm | |
| D30* | Benign neoplasm of the urinary organs | |
| D41* | Reproduction of insecure or unknown behaviour of the urinary organs | |
| F98.0* | Non-organic enuresis | |
| N02* | Recurrent and persistent hematuria | |
| N13* | Obstructive uropathy and refluxuroathy | |
| N16* | Tubulointerstitial kidney disease in other classified diseases | |
| N20* | Kidney and ureter stone | |
| N21* | Stone in the lower urinary tract | |
| N22-* | Urinary stone in other classified diseases | |
| N23 | Unspecified kidney colic | |
| N28* | Other diseases of kidney and ureter, not elsewhere classified | |
| N31* | Neuromuscular dysfunction of the urinary bladder, not classified elsewhere | |
| N32* | Other urinary bladder diseases | |
| N32.0 | Bladder neck obstruction | |
| N32.1 | Vesico intestinal fistula | |
| N32.2 | Urinary bladder fistula, not elsewhere classified | |
| N32.3 | Diverticulum of the urinary bladder | |
| N32.4 | Urinary bladder rupture, non-traumatic | |
| N32.8 | Other specified urinary bladder diseases | |
| N32.9 | Disease of the urinary bladder, unspecified | |
| N33* | Urinary bladder diseases in other classified diseases | |
| N36* | Other diseases of the urethra | |
| N36.0 | Urethral fistula | |
| N36.1 | Diverticulum of the urethra | |
| N36.2 | Urethral caruncle | |
| N36.3 | Prolapse of the urethral mucosa | |
| N36.8 | Other specified diseases of the urethra | |
| N36.9 | Diseases of the urethra, unspecified | |
| N37.8 | Other diseases of the urethra in diseases classified elsewhere | |
| N39.1 | Persistent proteinuria, unspecified | |
| N39.2 | Orthostatic proteinuria, unspecified | |
| N40 | Prostata hyperplasia | |
| N42* | Other diseases of the prostata | |
| N81.0 | Urethrocele in women | |
| N99* | Diseases of the urogenital system according to medical measures, not classified elsewhere | |
| N99.1 | Urethral stricture after medical measures | |
| Q64* | Other congenital malformations of the urinary system | |
| R30* | Pain when urinating | |
| R31 | Unspecified hematuria | |
| R32 | Unspecified urinary incontinece | |
| R33 | urinary retention | |
| R34 | Anuria and oligory | |
| R35 | Polyuria | |
| R36 | Urethral discharge | |
| R39* | Other symptoms affecting the urinary system | |
| Urinary stricture | | |
| N35* | Urethral stricture | |
| N99.1 | Urethral stricture after medical measures | |
| Urethral bleeding | | |
| N02* | Recurrent and persistent hematuria | |
| R31 | Unspecified hematuria | |
| Antibiotic resistance (combined with UTI see above) | | |
| U80!* | Gram-positive pathogens with certain antibiotic resistances that require special therapeutic or hygienic measures | |
| U81!* | Gram-negative pathogens with certain antibiotic resistances that require special therapeutic or hygienic measures | |
| Fever (combined with UTI see above) | | |
| R50* | Fever of other and unknown causes | |

| **Critical events: UTI/ other infection (see above) in combination with medication** | |
| --- | --- |
| **ATC** | **Description** |
| Use of antibiotics | |
| A07AA* | Antibiotics - intestinal |
| G01AA* | Antibiotic - gynaecological |
| J01* | Antibiotics for systemic use |
| J01XX05 | Methenamine |
| Use of prophylactic antibiotics | |
| J01DB01 | Cephalexin |
| J01EA01 | Trimethroprim |
| J01EE01 | Sulfamethoxazol und Trimethroprim |
| J01MA02 | Ciprofloxacin |
| J01MA06 | Norfloxacin |
| J01XE01 | Nitrofurantoin |
| J01XE51 | Nitrofurantoin, combinations |

| **Critical events: UTI in combination with procedures** | |
| --- | --- |
| **EBM** | **Description** |
| Urologic procedures | |
| 01732 | Early detection of diseases according to health screening guidelines |
| 08310 | Apparative examination of a patient with urinary incontinence |
| 08311 | Urethro(-cysto)scopy |
| 08312 | Supplement to fee item 08311 for transurethral therapy with botulinum toxin |
| 08313 | Supplement to fee schedule item 08312 for the observation of a patient following transurethral therapy with botulinum toxin |
| 26310 | Urethro(-cysto)scopy of the man |
| 26311 | Urethro(-cysto)scopy of the woman |
| 26312 | Urethra pressure profile measurement with continuous registration |
| 26313 | Additional lump-sum examination for urinary incontinence or neurogenic bladder voiding disorder |
| 26316 | Supplement to fee schedule items 26310 and 26311 for transurethral therapy with botulinum toxin |
| 26317 | Supplement to fee schedule item 26316 for the observation of a patient following transurethral therapy with botulinum toxin |
| 32030 | Orienting examination |
| 32031 | Microscopic examination of urine for morphological components |
| 32033 | Urine strip test |
| 32052 | Quantitative determination(s) of morphological constituents by Chamber counting of the cells in the urine collection, also in several fractions within 24 hours (Addis-Count) |
| 32720 | Urine examination with at least two culture media (except immersion culture media) and/or with growth measurement by apparatus |
| 32880 | Laboratory lump sum for examinations in connection with the provision of fee item 01732 (health examination) using a test strip |

**Codes used in table 3**

| **Medication (at least one prescription)** | |
| --- | --- |
| **ATC** | **Description** |
| Medication for functional disorders of the bladder | |
| A03AA* | Synthetic anticholinergics, esters with tertiary amino groups |
| A03AB* | Synthetic anticholinergics, quaternary ammonium compounds |
| C01CE* | Phosphodiesterase inhibitors |
| C03* | Diuretics |
| C08* | Calcium channel blockers |
| C09A* | ACE inhibitors, pure |
| C09B* | ACE inhibitors, combinations |
| G03C* | Estrogen |
| G03CD* | Estrogen, vaginal preparations |
| G04B* | Urologics |
| G04BA* | Urine-acidifying agent |
| G04BC* | Urinary concrement solvents |
| G04BD* | Agent for frequent bladder emptying and urinary incontinence |
| G04BE* | Agent for erectile dysfunction |
| G04BH* | Homeopathic and anthroposophic urological medicines |
| G04BP* | Herbal urologics |
| G04BX* | Other urologics |
| G04C* | Agent for benign prostatic hyperplasia |
| Use of antidepressants | |
| N06A* | Antidepressants |
| N06AX21 | Duloxetine |
| N06B* | Psychostimulants, drugs for ADHD and nootropics |
| Use of sleep aids | |
| N05C* | Hynotics and sedatives |
| Use of muscle relexans | |
| M03* | Muscle relexants |
| Use of antibiotics | |
| A07AA* | Antibiotics - intestinal |
| G01AA* | Antibiotic - gynaecological |
| J01* | Antibiotics for systemic use |
| J01XX05 | Methenamine |
| Use of prophylactic antibiotics | |
| J01DB01 | Cephalexin |
| J01EA01 | Trimethroprim |
| J01EE01 | Sulfamethoxazol und Trimethroprim |
| J01MA02 | Ciprofloxacin |
| J01MA06 | Norfloxacin |
| J01XE01 | Nitrofurantoin |
| J01XE51 | Nitrofurantoin, combinations |
| Use of supplements & herbal anti-infectives | |
| A11* | Vitamins |
| A11G* | Ascorbic acid (vitamin C), incl. Combinations |
| A12* | Minerals |
| B05BC* | Osmodiuretics |
| B05BC01 | Mannitol |
| B05BC51 | Mannitol, combinations |
| G04BA* | Urinary acidifying agents |
| G04BA04 | L-Methionine |
| G04BP50 | Other herbal urologics, combinations |
| G04BP01 | Bearberry leaves |
| G04BX20 | Escherichia coli (URO-Vaxom) |
| Use of pain medication | |
| N02* | Analgesics |
| N02A* | Opioids |
| Sterile rinsing of bladder (rinsing solution) | |
| B05C* | Rinsing solutions |
| B05CA* | Anti-infectives |
| B05CX04 | Mannitol |

| **Medical aid number.** | **Description** |
| --- | --- |
| **15.25.14*** | **Single-use catheter for ISC** |

**Codes used in table 4**

| **Hospitalization causes (specific inpatient primary diagnosis)** | |
| --- | --- |
| **ICD-10 GM** | **Description** |
| UTI (Urinary tract infections) | |
| N30.0 | Acute cystitis |
| N30.1 | Interstitial cystitis (chronic) |
| N30.2 | Other chronic cystitis |
| N30.9 | Cystitis unspecified |
| N34.1 | Unspecific urethritis |
| N34.2 | Other urethritis |
| N37.0 | Urethritis in diseases classified elsewhere |
| N39.0 | Urinary tract infection, localization not further described |
| Urologic diseases | |
| C61 | Malignant neoplasm of the prostate gland |
| C66 | Malignant neoplasm of the ureter |
| C67* | Malignant neoplasm of the urinary bladder |
| C68* | Malignant neoplasm of other and unspecified urinary organs |
| D29.1 | Benign prostatic neoplasm |
| D30* | Benign neoplasm of the urinary organs |
| D41* | Reproduction of insecure or unknown behaviour of the urinary organs |
| F98.0* | Non-organic enuresis |
| N02* | Recurrent and persistent hematuria |
| N13* | Obstructive uropathy and refluxuroathy |
| N16* | Tubulointerstitial kidney disease in other classified diseases |
| N20* | Kidney and ureter stone |
| N21* | Stone in the lower urinary tract |
| N22-* | Urinary stone in other classified diseases |
| N23 | Unspecified kidney colic |
| N28* | Other diseases of kidney and ureter, not elsewhere classified |
| N31* | Neuromuscular dysfunction of the urinary bladder, not classified elsewhere |
| N32* | Other urinary bladder diseases |
| N32.0 | Bladder neck obstruction |
| N32.1 | Vesico intestinal fistula |
| N32.2 | Urinary bladder fistula, not elsewhere classified |
| N32.3 | Diverticulum of the urinary bladder |
| N32.4 | Urinary bladder rupture, non-traumatic |
| N32.8 | Other specified urinary bladder diseases |
| N32.9 | Disease of the urinary bladder, unspecified |
| N33* | Urinary bladder diseases in other classified diseases |
| N36* | Other diseases of the urethra |
| N36.0 | Urethral fistula |
| N36.1 | Diverticulum of the urethra |
| N36.2 | Urethral caruncle |
| N36.3 | Prolapse of the urethral mucosa |
| N36.8 | Other specified diseases of the urethra |
| N36.9 | Diseases of the urethra, unspecified |
| N37.8 | Other diseases of the urethra in diseases classified elsewhere |
| N39.1 | Persistent proteinuria, unspecified |
| N39.2 | Orthostatic proteinuria, unspecified |
| N40 | Prostata hyperplasia |
| N42* | Other diseases of the prostata |
| N81.0 | Urethrocele in women |
| N99* | Diseases of the urogenital system according to medical measures, not classified elsewhere |
| N99.1 | Urethral stricture after medical measures |
| Q64* | Other congenital malformations of the urinary system |
| R30* | Pain when urinating |
| R31 | Unspecified hematuria |
| R32 | Unspecified urinary incontinece |
| R33 | urinary retention |
| R34 | Anuria and oligory |
| R35 | Polyuria |
| R36 | Urethral discharge |
| R39* | Other symptoms affecting the urinary system |
| Other infections | |
| A39.2 | Acute meningococcal sepsis |
| A39.3 | Chronic meningococcal sepsis |
| A39.4 | Meningococcal sepsis, unspecified |
| A40* | Streptococcal sepsis |
| A41* | Other Sepsis |
| A48.8 | Other specified bacterial diseases |
| A49* | Bacterial infection of unspecified localization |
| B37.4 | Candidosis at other sites of the urogenital system |
| B37.7 | Candida sepsis |
| B95-*! | Streptococci and staphylococci as causes of diseases classified in other chapters |
| B96-*! | Other specified bacteria as the cause of diseases classified in other chapters |
| B96.2! | Escherichia coli [E. coli] and other enterobacteriaceae as cause of diseases classified in other chapters |
| J12* | Virus pneumonia, not elsewhere classified |
| J13* | Pneumonia caused by Streptococcus pneumoniae |
| J14* | Pneumonia caused by Haemophilus influenzae |
| J15* | Pneumonia caused by bacteria, not classified elsewhere |
| J16* | Pneumonia caused by other infectious agents, not elsewhere classified |
| J17* | Pneumonia in diseases classified elsewhere |
| J18* | Pneumonia, pathogen not described in more detail |
| I33* | Acute and subacute endocarditis |
| N45* | Orchitis and epidimytitis |
| R50* | Fever of other and unknown causes |
| R65.-* | Systemic inflammatory response syndrome [SIRS] |
| U80!* | Pathogens with certain antimicrobial resistance requiring special therapeutic or hygienic measures |
| U80!* | Gram-positive pathogens with certain antimicrobial resistance requiring special therapeutic or hygienic measures |
| U81! | Bacteria with multiresistance to antibiotics |
| U81!* | Gram-negative pathogens with certain antimicrobial resistance requiring special therapeutic or hygienic measures |

| **Physician visits (outpatient contact)** | | |
| --- | --- | --- |
| **AGS** | **physician group (referring Table 4)** | **cluster** |
| 01 | General practitioners (GP) | GP |
| 02 | Practical doctor (GP) | GP |
| 03 | Internal specialist (GP) | GP |
| 34 | Paediatrician (GP) | GP |
| 51 | Neurology and psychiatry | Psych |
| 53 | Neurology and psychiatry | Psych |
| 58 | Psychaitrie and psychotherapy | Psych |
| 61 | Psychotherapy (medical doctor) | Psych |
| 67 | Urology |  |
| 68 | Psychological psychotherapist | Psych |

**Codes used in table 5**

| **Costs for medical aids and remedies** | |
| --- | --- |
| **Medical aid number** | **Description** |
| Other catheters | |
| 15.25.14* | ISC catheters |
| 15.25.15* | Baloon catheters |
| 15.25.04* | External urine traps |
| 15.25.04.1 | Urine trap for women |
| 15.25.04.2 | Urine trap for men |
| 15.25.04.3 | Urine trap for children |
| 15.25.04.4 | Urinal condom/roller funnel, containing latex, not ready to use |
| 15.25.04.5 | Urinal condom/roller funnel, containing latex, ready to use |
| 15.25.04.6 | Urinal condom/roller funnel, made of latex-free materials, not ready to use |
| 15.25.04.7 | Urinal condom/roller funnel, made of latex-free materials, ready to use |
| 15.25.04.8 | Urinal condom/roller funnel at ISC, special shape |
| 15.25.20* | Intraurethral incontinence therapy systems |
| 15.25.22* | Special catheters for therapy |
| 15.25.22.0 | Catheter for instillation |
| 15.25.22.1 | Catheter for self-dilatation |
| Other medical aids | |
| 15.25.30* | Absorbent incontinence pads |
| 15.25.31* | Absorbent incontinence pants (not reusable) |
| 19.99.01* | Disposable gloves |

| **Costs for medication** | |
| --- | --- |
| **ATC Code** | **Description** |
| Use of antibiotics (referring Table 5) | |
| A07AA* | Antibiotics - intestinal |
| G01AA* | Antibiotic - gynaecological |
| J01* | Antibiotics for systemic use |
| J01XX05 | Methenamine |
| Use of pain medication | |
| N02* | Analgesics |
| N02A* | Opioids |

| **Costs for UTI (inpatient sector)** | |
| --- | --- |
| **ICD-10** | **Decription** |
| UTI (Urinary tract infections) (referring table 5) | |
| N30.0 | Acute cystitis |
| N30.1 | Interstitial cystitis (chronic) |
| N30.2 | Other chronic cystitis |
| N30.9 | Cystitis unspecified |
| N34.1 | Unspecific urethritis |
| N34.2 | Other urethritis |
| N37.0 | Urethritis in diseases classified elsewhere |
| N39.0 | Urinary tract infection, localization not further described |
